# Influence of nitric oxide in the improvement of muscle power

**DOI:** 10.1590/1413-785220152306148249

**Published:** 2015

**Authors:** Daniela Navarro D'Almeida Bernardo, Flávio Fernandes Bryk, Patrícia Maria de Moraes Barros Fucs

**Affiliations:** 1 Centro Universitário Católico Salesiano - UniSALESIANO, Araçatuba, SP, Brazil.; 2 Irmandade da Santa Casa de Misericórdia de São Paulo, São Paulo, SP, Brazil; 3 Faculdade de Ciências Médicas da Santa Casa de São Paulo (FCMSCSP), Department of Orthopedics and Traumatology, São Paulo, SP, Brazil

**Keywords:** Nitric oxide, Exercise therapy, Muscle strength, Dietary supplements, Physical education and training

## Abstract

**OBJECTIVE:**

To evaluate whether nitric oxide (NO) supplementa-tion is directly related to increased muscle power in response to strength exercise training

**METHODS:**

The study included 36 individuals who underwent training for eight weeks (three times per week) with weights, who were randomly divided into two groups, both receiving the same training protocol, but one group used 3g of arginine, as a precursor of NO, and the other received placebo

**RESULTS:**

There was no significant difference between groups, only a significant difference for both groups between moments: before and after the training protocol

**CONCLUSION:**

Oral administration of arginine asso-ciated with a training program did not increase the muscular power of individuals. **Level of Evidence I, Study Type: Highquality randomized trial with statistically significant diffe-rence or no statistically significant difference but narrow confidence intervals.**

## INTRODUCTION

Nitric oxide (NO) is one of the smallest and simplest molecules ever biosynthesized.^1^ It is a free radical in form of a colorless gas, with seven electrons of nitrogen and eight electrons of oxygen, with one unpaired electron.^2^

NO is an endogenous molecule involved in numerous physiolo-gical processes ranging from neurotransmission to modulation of the inflammatory state, acting in the aggregation, vasodilation and chemotaxis. NO also has bactericidal properties.^3^ ^5^ NO is one of the most important mediators of intra-and extracellular processes.^6^ Because it has a vasodilator function, NO acts in situations of muscle overload, stimulating the transition between the muscle fiber types.^7^

The NO produced by vascular endothelial cells is an important regulator of the vascular function.^8^ The production of NO in humans occurs when L-arginine is converted to L-citrulline, in a reaction catalyzed by the enzyme nitric oxide synthase (NOS).^7^ The NO produced by endothelial cells plays an essential role in the relaxation of the blood vessel. In pathophysiological conditions, vascular relaxation occurs when the membrane receptors of endothelial cells are activated by soluble stimu-lators or when there is an increase in the friction exerted by circulating cells on the endothelial layer, leading to activation of the enzyme e-NOS (endothelial NO synthase) present in these cells and consequent NO production.^9^ The e-NOS is strategically anchored to the endothelial cell membrane, which favors the presence of large amounts of NO near the muscle layer of the vessel and next to circulating blood cells. The NO produced in the endothelial cell diffuses rapidly into the muscle cells and to the vascular lumen. The rapid diffusion and the ease with which this molecule penetrates into other cells, thanks to its small size and its lipophilicity, are crucial to its biological roles.^10^

According to Cerqueira and Yoshida,^11^ NO mediates several phenomena, such as endothelium-dependent vasorelaxation, platelet adhesion and aggregation, and baseline blood pres-sure regulation. In blood vessels, NO modulates vascular dia-meter and vascular resistance through its ability to relax the vascular smooth muscle.

Exercise training improves cardiovascular autonomic function and endothelial vasodilator systemically, promoting beneficial cardiac and vascular effects. These benefits may be also rela-ted to the increased production of NO.^12^

According to Flakoll et al.,^13^ supplementation of arginine (with beta-hydroxy-beta-methylbutirate) improves contractile force by increasing muscle protein synthesis. For these authors, supplementation for long periods, concomitant with a resis-tance-training program, may be associated with improvement of contractile force by increasing muscle protein synthesis. Apparently, the oral administration of the supplement provides better quality of training by three interrelated and interdepen-dent mechanisms, triggered simultaneously by vasodilation: increased blood perfusion, facilitating the supply of oxygen and nutrients to the tissues,^14^ ^15^ and greater glucose supply, with higher energy substrate for muscle contraction.^16^ Ac-cording to Angeli et al.,^7^ the improved perfusion of skeletal muscle itself can contribute to a better quality of the resistance training, with increasing effects of training in muscle mass and in contractile power over time.

Definitions of strength and power are often contradictory and confusing. Nevertheless, according to the physics laws, streng-th or force (F) is expressed as the product of mass and acce-leration (F [N] = m [kg]). a [m/s^2^]).^17^ Power (P) represents the mechanical work under a variety of conditions, and it can be characterized as follows: [P (W) = F (N) x V (m.s^-1)^]. Therefore, muscle power is highly dependent on the strength and both are important for sports performance and everyday activities.^17^ Angeli et al^7^ found that, after two months of weight training, a group that received supplementation with arginine had signi-ficantly higher weight, lean mass and lower limb strength and significantly lower body fat percentage, while the control group showed no significant differences for the same period. As pro-longed administration of arginine increases NO production, its supplementation has been associated with improved contractile function of the skeletal muscle.

There is a lack of information and studies on the effect of sup-plementation with NO and anaerobic exercises in the improve-ment of muscle power in humans.

The objective of the present study is to investigate the effects of supplementation with NO in muscle power after performing an exercise protocol for muscle strengthening. The hypothesis would be that such supplementation would lead to improve in muscle strength. Our goal is also to evaluate whether sup-plementation of arginine combined with muscle strengthening exercises is directly related to the increase of muscle power in horizontal and vertical jump functional tests.

## METHODS

This is a randomized, placebo-controlled, double blind clinical trial. The study was performed at the Physical Therapy Reha-bilitation Service of Santa Casa de Misericórdia de São Paulo, a university hospital, after approval by the local Research Ethics Committee, under number 267152. The study used a convenience sample of Physical Therapy graduated students. Every participant was informed about the research objectives and methods and signed a Free and Informed Consent forms for participation.

The study recruited all 44 graduate students in the Physical Therapy Department (20 men and 24 women) as volunteers. They were recruited personally in the classroom. They ought to be aged 20-30 years old to be included, performing no athletic activity, and without any pulmonary or cardiorespiratory disease. Exclusion criteria were: refusal to participate in examinations or in physical training sessions, refusal to receive or presenting gastrointestinal problems preventing the administration of pla-cebo or the NO precursor arginine (orally administrated), pre-sence of fractures, recent surgical treatment, orthopedic disease in the last six months and history of cardiovascular disease (participants were asked about any heart disease, hypotension episodes or hypertension diagnosis or current treatment, as these would also constitute exclusion criteria).

We registered demographic data such as name, age and con-tact phone number. All participants were weighed and mea-sured for height in a recently calibrated scale with maximum weight of 150 kg and maximum height of 2.20m.

Of the 44 regular students in the Department, 36 (16 women and 20 men) participated in the study with full documentations and were present in all examinations. All subjects had no history of physical activity prior to the study. Participants were aged 21-30 years old (mean 23.97 years old, standard deviation, SD, 2.38 years old), and body weight between 46 and 135 kg (mean 72.90 kg, SD 17.69), height between 145 and 184 cm (average 1.66 m, SD 0.09) and body mass index (BMI) between 18.53 and 42.13 kg/m^2^ (average 25.87 kg/m^2^, SD 4,59).

The subjects were divided into two groups, both participating in the exercising protocol; the study group receiving arginine su-pplementation (that we called "the NO group") and the control group (CG) receiving maltodextrin, as a placebo. Maltodextrin is a complex carbohydrate derived from the hydrolysis of corn, which does not interfere with the study results, since the amount of maltodextrin was not sufficient to optimize the workout perfor-mance. Both arginine and placebo were produced by the same laboratory ( *Athlética Laboratório* ADS). Arginine and placebo were orally administrated in equal capsules, both in the amount of 0.5g/capsule. A total of six capsules per training day was gi-ven to the participants, totaling 3 g of arginine or placebo, which was administered 45 minutes before training, with a previous period of one hour fasting for better effects.

One researcher (FFB) was responsible for the randomization, which was conducted by using sealed opaque envelopes. Allo-cation of participants was hidden from the other researchers and from trial participants.

### Assessment Protocol

Muscle power was assessed twice in the study: the first proto-col before muscle strengthening exercising protocol (M1), and the second eight weeks after the beginning of the exercising protocol (M2). In order to evaluate the effects of supplemen-tation, the groups underwent two functional tests; the first was the single hop test,^18^ which is used to assess the muscle power of the lower limbs. This test consists of jumping forward with one leg, hands behind the back, reaching the maximum distance, which was registered. The test was performed twice with the dominant and the non dominant leg, the first for the participant to understand and adjust the movement and the second for analysis.

The second functional test, the vertical jump,^18^ aims to me-asure the power of the lower limbs in the vertical plane. The participant stands beside a wall, keeping the soles of his feet in contact with the ground. The test consists of jumping as high as possible, flexing the legs and swinging arms, if necessary, to perform the jump. The highest point reached is marked on the wall and height is measured. The test was performed twice: the first jump for the participant to understand the movement and the second jump for analysis.

The distance and height obtained with the single hop and the vertical jump tests were normalized by the height of each indi-vidual by the formula:

Test distance or height/individual height x 100.

### Training protocol

The training protocol was composed of exercises three times a week totaling 24 sessions, as described in Table 1. The total time for performing the training protocol was 30 min, with an interval of rest between sessions of 1 min. The load set for mus-cle strengthening was: starting the exercises without weight and increasing 1 kg every two weeks in a row, except knee exten-sion, in which 70% of the maximum resistance (MR) was used. Aiming to reduce the margin of error of the exercise protocol, strategies were adopted for accurate understanding of the mo-vements by participants, such as: standardized instructions were provided before performing the exercises, so that the par-ticipant was aware of the routine involved, the participant was instructed on the technique of the exercise; the evaluator was watchful for the body positioning by the participant, because small variations in the positioning of the joints involved in the movement could result in the use of other muscles in the task, leading to wrong interpretations of study results.

**Figure 1:**
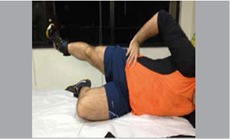
Abduction.

**Figure 2:**
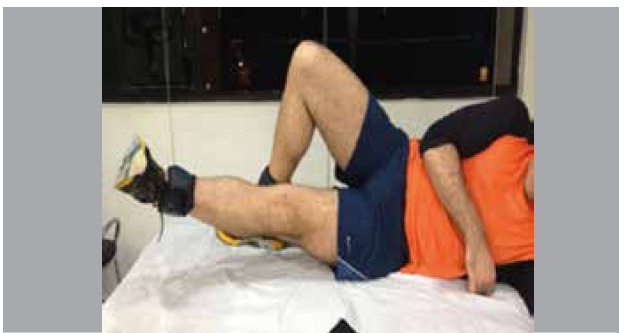
Adduction.

**Figure 3:**
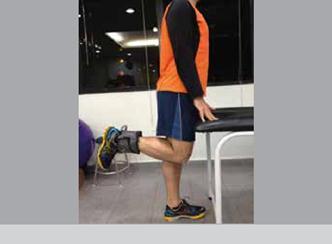
Knee flexion.

**Figure 4:**
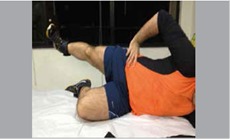
Knee extension.

**Figure 5:**
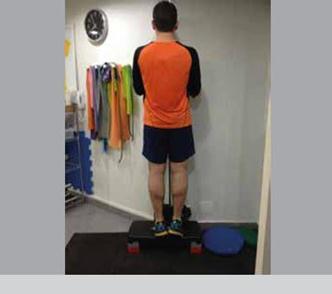
Bipodal calf.

**Figure 6.**
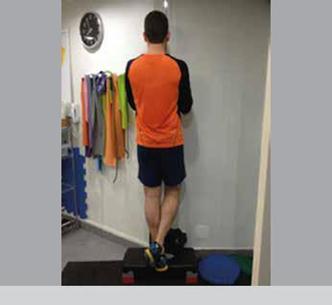
Unipodal calf.

The exercises of the training protocol aimed at strengthening the abductor and adductor muscles of the hip, knee flexors and extensors and foot plantar flexors are shown in Figures 1 to 6. Four sets of 10 repetitions of each exercise were performed.

**Table 1:**
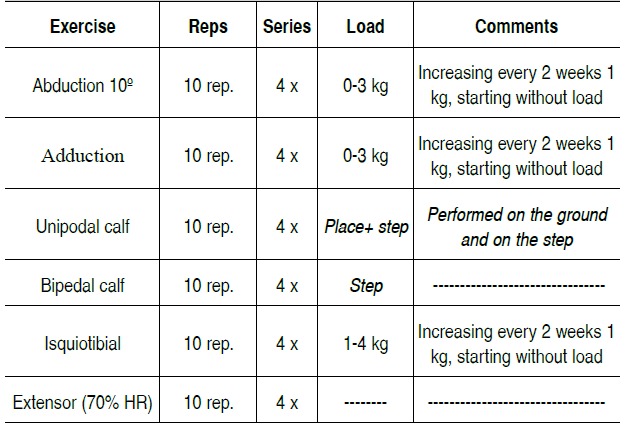
. Training program (exercising 3 times per week for 2 months, with 8 weeks of training in total).

### Statistical analysis

All data were recorded in Microsoft Office Excel 2007 sheets, and copied to the program SPSS version 20.0, used for sta-tistical analysis. To compare the two groups with regard to the homogeneity of data such as body weight, height, body mass index (BMI) and age at the beginning of the study, we used the analysis of variance (ANOVA) test of independent measu-rements. For intra groups comparisons at the beginning and end of the intervention and inter groups comparisons after the intervention, ANOVA test for repeated measures was used. The initial calculation of the sample was based on the comparison of means using as reference similar studies, considering mean differences of 1.5 and standard deviation of 2, with the level of significance of 5% and a statistical power of 80%.

## RESULTS

Table 2 shows that there was no significant difference in age, weight and BMI at baseline, but there was a statistical difference in the sample in height, with the group receiving arginine per-forming a greater height than the placebo group.

In the single hop test, normalized by height, there was a signifi-cant difference inside the groups comparing baseline and final values, but there was no significant difference between groups, according to Table 2.

For the vertical jump test, there was also a significant difference comparing baseline and final values, but there were no signifi-cant differences between groups, according to Table 2.

**Table 2:**
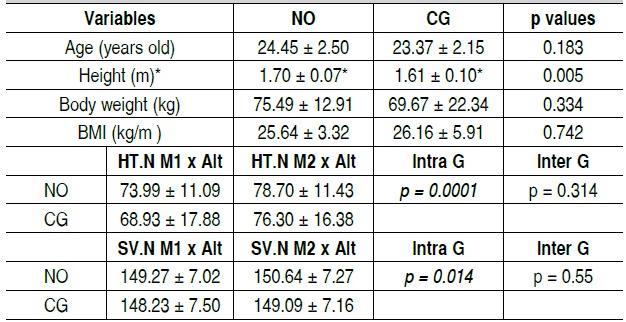
Mean and standard deviation of baseline values for age, hei-ght, body weight and body mass index (BMI) and for the study results of height normalized single hop test and height normalized standard vertical jump for the groups receiving nitric oxide supplementation (NO) and the control group (CG)

*Statistically significant difference, p≤ 0.05; HT.N M1 x Alt: height normalized single hop test value at baseline; HT.N M2 x Alt: height normalized single hop test value at the final moment; SV.N M1 x Alt; height normalized standard vertical jump at baseline; SV.N M2 x Alt; height normalized standard vertical jump at the final moment.

## DISCUSSION

The literature has shown that muscle strength and power are essential both for physical performance and for health.^17^ ^19^ ^20^ For sports training, muscle power is one of the most important variables.^21^ ^22^ This is also true for everyday activities, in which the muscle power plays an important role.^17^ ^19^

Among the several studies about NO and muscle mass and strength gains, the study by Angeli et al.^7^ stands out. The au-thors concluded that oral administration of 3 g/day of a NO precursor potentiates the effects of weight training, providing greater strength gains and muscle mass; however, the asso-ciation of supplementation and exercise for a possible impro-vement of muscle power is not elucidated in the literature, there are no studies proving that using the supplement leads to the increase of muscular power. Since the variable power is direc-tly proportional to the variable strength and speed, if there is an improvement in muscle strength, consequently we should expect improvements in muscle power.

In order to evaluate the improvement of muscle power, studies have used specific tests, such as the vertical jump and single hop test.^23^ ^25^ According to Hespanhol et al.,^23^ peak muscle po-wer, average power and fatigue index are reliable measures with regard to repeated measures in assessing the vertical jump test. Among other methods designed to provide information on muscle function, there is the single hop test described by Daniel et al.,^18^ which evaluates the components of strength and power. For the stability of a damaged muscle segment, the method of Selistre et al^26^ was added. This method has well-established advantages, such as low cost, easy application, good reliability and validity.^27^ ^29^ The distance measured in the jumps of the functional tests was normalized with the participant height to demonstrate that there is no significant difference between the groups, even considering that the participants in one group were higher than the others', as shown in Table 2. One limitation of this study was that there was the difference in height between groups, a fact that could influence the distance and height found the single hop test and vertical jump, respectively. However, the results were normalized by the height of each participant, minimizing, thus this bias.

 In the present study, there was no significant difference be-tween groups, but there was a significant difference in both groups comparing baseline and final evaluation. Based on the results obtained, with the difference between the two mo-ments, it is possible to say that the supplementation of argi-nine, as a precursor of NO, was not effective for improving muscle power, but it was able to improve muscle strengthe-ning exercising. Unfortunately, there are no literature data that could be compared with the findings of this research, since the great majority of studies report the cardioprotective and vasodilators effects of NO, as well as improvements in muscle strength and lean body mass.

Regarding the association of strength, muscle mass and muscle power, what differentiates strengthening exercises and strength and muscle power is the rate of speed for the execution of the movement.^30^ ^32^ Force is usually related to work involving heavy loads, without the need of imposing high-speed movement. Power, on the other hand, has already been associated with loads below those recommended for strength, being more clo-sely related to the speed of movement than to the load itself.^32^ There are two variables in the equation to obtain the calculation of muscle power, strength and speed. In this study, the force ratio was the variable recommended for the training protocol, since there are studies emphasizing the improvement of muscle strength using the NO precursor,^15^ ^16^ but speed ratio was not used in this protocol, and this could have been the reason for not improving muscle power between the groups. Therefore, the question about the effects of the combination of NO precursor and muscle power has still to be investigated in the future.

A limitation of this study was the use of single hop and vertical jump tests. Single hop was designed to assess knee stability, and we believe that it might proxy performance, because an unstable knee could theoretically indicate a weak muscle in that limb. Vertical jump is used to assess muscle power. However, since the studies in the literature that address muscle strength use these tests, and in order to provide homogeneous data for comparison, we opted to use them as well.

## CONCLUSION

Supplementation of arginine (3 g/training day) in healthy indivi-duals after performing muscle strengthening exercises did not improve muscle power. 
